# Bioactivity and health effects of garlic essential oil: A review

**DOI:** 10.1002/fsn3.3253

**Published:** 2023-02-07

**Authors:** Lei Huang, Zhenxin Liu, Jing Wang, Jiaolong Fu, Yonglu Jia, Lilian Ji, Taoyun Wang

**Affiliations:** ^1^ School of Chemistry and Life Sciences Suzhou University of Science and Technology Suzhou China; ^2^ Department of Stomotology, Suzhou Kowloon Hospital Shanghai Jiaotong University School of Medicine Suzhou China

**Keywords:** bioactive component, biological activity, essential oil, garlic, health function

## Abstract

Garlic (*Allium sativum* L.), the underground bulb of the *Allium* plant in the family Liliaceae, is a common and popular spice that has historically been used to prevent and treat many different diseases such as pain, deafness, diarrhea, tumors, and other healthy problems. Garlic essential oil contains a variety of organosulfur compounds, such as the most representative diallyl disulfides (DADS) and diallyl trisulfides (DATS), which have attracted great interest in medicine, food, and agriculture because of their rich biological activities. This paper reviews the research progress on the composition and bioactivities of garlic essential oil mixtures and the bioactivity of some typical monomeric sulfides in garlic essential oil. The active mechanisms of representative sulfides in garlic essential oil were analyzed, and the applications of garlic essential oil in functional food, food additives, and clinical treatment were discussed. Combined with the current research status, the limitations and development direction of garlic essential oil in the study of molecular mechanism were discussed, which is of great significance to the development of garlic essential oil as a natural and safe alternative medicine for treatment.

## Graphical abstract

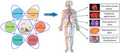

## 1 INTRODUCTION

At present, natural products as effective therapeutic agents for human diseases are receiving extensive attention, among which plant‐derived natural products are the representative (Alinezhad et al., 2013). Plant‐derived natural products mainly refer to the secondary metabolites of plants, which widely exist in nature, have extremely rich types, and have many types of effective antibacterial components, such as essential oils, polyphenols, flavonoids, alkaloids, and organic acids. Because of their efficacy and a variety of antibacterial mechanisms to prevent the development of drug resistance. Essential oils are aromatic oily liquids, which are known as complex mixtures of several volatile constituents including sesquiterpenes, monoterpenes, aldehydes, alcohols, esters, and ketones. Research shows that essential oil plays an important role in the process of plants dealing with pests, fungi, and bacteria (Harkat‐Madouri et al., 2015). Therefore, the use of plant essential oil for drug development is very important for the prevention and treatment of various diseases caused by pathogenic microorganisms.

Garlic (*Allium sativum* L.) is an underground bulb of *Allium* in Liliaceae. It originated in Central Asia and the Mediterranean and was introduced into Japan and South Asia in the 9th century. And now that it has been widely cultivated all over the world. Garlic is a famous dual‐use plant for food and medicine. It has been widely used in the treatment and prevention of headache, tumor, diarrhea, and other diseases since ancient times (Nagini, 2008). Due to its good pharmacological activity, garlic has become popular for applications in chemoprevention and chemotherapy research. For example, in the field of food, previous studies reported that nano‐phytosomes prepared from garlic essential oil can be used as food preservatives (Nazari et al., 2019). Garlic contains more than 33 different organic sulfur compounds, which are responsible for its specific flavor and pharmacological effects (De Greef et al., 2020). Previous studies have shown that 100 g of fresh garlic contains 4.4 g of protein, 0.2 g of fat, 23 g of carbohydrate, 0.7 g of crude fiber, and 1.3 g of ash (Zhang, Bai, et al., 2020; Zhang, Liu, et al., 2020). At present, many bioactive compounds have been found in garlic essential oil. In addition to organic sulfides such as DADS and DATS, garlic essential oil is also very rich in steroidal saponins, essential amino acids, phenolic compounds, saponin ligands, and other non‐sulfur compounds (Amagase, 2006).

**FIGURE 1:**
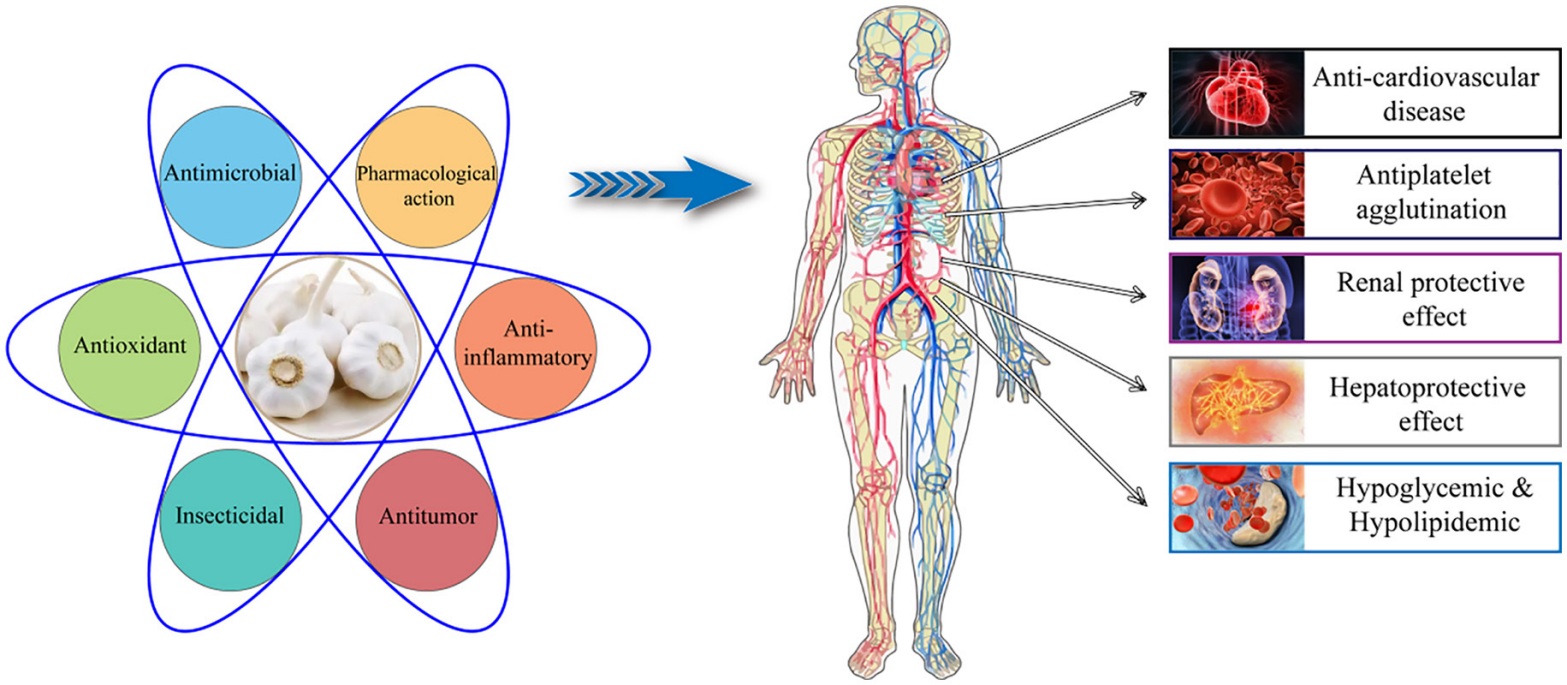
Biological activity of garlic essential oil and its effect on human health.

The representative volatiles in garlic essential oil are allyl sulfides such as DADS and DATS. At present, there is little review on the bioactivities of garlic essential oil. Therefore, this article mainly introduces the components, properties, and related applications of garlic essential oil. In addition, this article also systematically reviewed the antibacterial, insecticidal, antioxidant, and other biological activities of garlic essential oil and its effects on human health, such as anti‐tumor, hypoglycemic, and anti‐inflammatory (Figure 1).

## 2 CHEMICAL COMPOSITION OF GARLIC ESSENTIAL OIL AND ITS BIOSYNTHETIC PATHWAY

Garlic essential oil contains more than 95% sulfides and mostly allyl polysulfides, of which the five most important allyl sulfides are diallyl sulfide (1.9%–9.5%), diallyl disulfide (20.8%–27.9%), diallyl trisulfide (16.8%–33.4%), allyl methyl disulfide (4.4%–8.3%), and allyl methyl trisulfide (14.5%–19.2%) (Satyal et al., 2017). It was found that a large amount of γ‐glutamylcysteine in garlic bulbs was oxidized to an inactive derivate‐alliin by the action of alliinase released from the vesicles during garlic crushing, which was further metabolized to the highly active thiosulfite diallyl ester and finally formed allicin. Allicin can be rapidly broken down into many sulfur‐containing compounds, including DADS and DATS. Until now, there are about 68 different compounds have been identified in garlic essential oil (Table 1).

**TABLE 1:** Compounds identified in garlic essential oil.

Number	Compound	Structure	References
1	Prop‐2‐ene‐1‐thiol	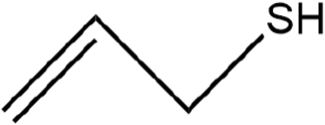	(Edris & Fadel, 2002; Rao et al., 2006)
2	Allyl(methyl)sulfane	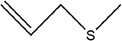	
3	(*E*)‐but‐2‐enal	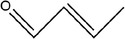	
4	2‐Methylpent‐4‐enal	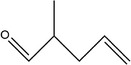	
5	1,2‐Dimethyldisulfane	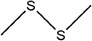	(Abu‐Lafi et al., 2004; Ichikawa et al., 2006)
6	(*E*)‐2‐methylbut‐2‐enal	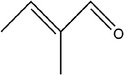	(Edris & Fadel, 2002)
7	2‐Methylenepentanal	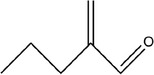	(Dziri et al., 2013; Zhang, Yang, et al., 2012)
8	(*E*)‐pent‐2‐enal	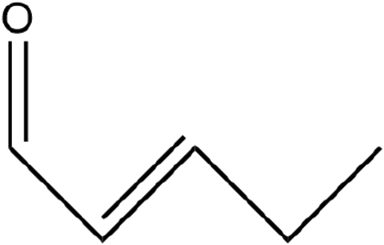	
9	1,2‐Diallyldisulfane	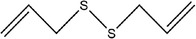	
10	Diallylsulfane	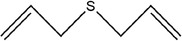	
11	2‐Methylfuran	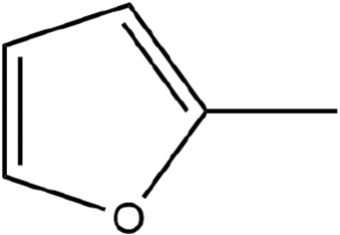	(Zhang, Yang, et al., 2012)
12	2,4‐Dimethylthiophene	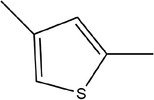	
13	3‐Methylpyridine	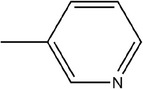	
14	2,5‐Dimethylpyridine	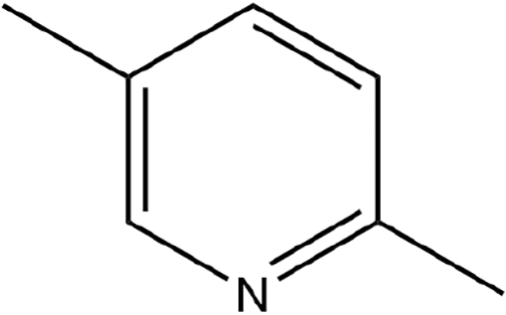	
15	(2 *E*,4 *E*)‐hexa‐2,4‐dienal	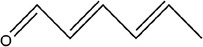	
16	*O*‐allyl ethanethioate	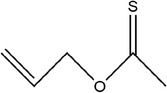	(Schulz et al., 1998)
17	2‐Ethylidene‐1,3‐dithiane	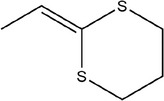	(Ichikawa et al., 2006)
18	2‐Vinyl‐1,3‐dithiolane	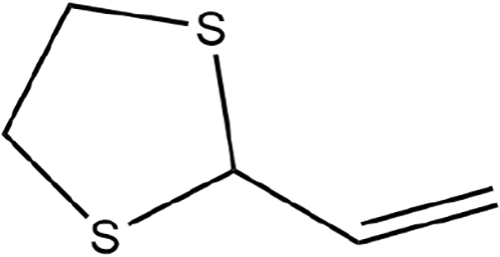	
19	Thiophene‐2‐carbaldehyde	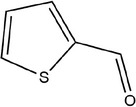	
20	3,5‐Diethyl‐1,2,4‐trithiolane	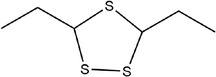	
21	1,3,5‐Trithiane	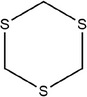	
22	(*E*)‐2‐(prop‐1‐en‐1‐ylthio)thiophene	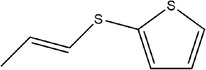	
23	Methyl 2‐(thiophen‐2‐yl)acetate	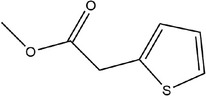	
24	4,5‐Dimethylisothiazole	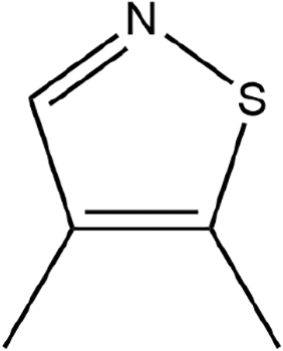	
25	1,3‐Phenylenedimethanethiol	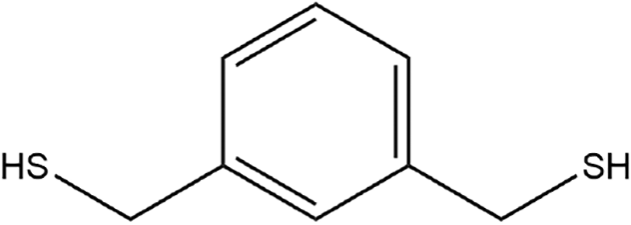	
26	Benzo[*b*]thiophene	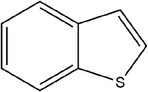	
27	*S*‐allyl prop‐2‐ene‐1‐sulfinothioate	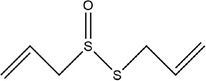	(Kim, Chun, et al., 2011; Kim, Park, et al., 2011; Lanzotti, 2006)
28	(*E*)‐1‐allyl‐2‐(3‐(allyl(methylene)‐λ^4^‐sulfaneyl)prop‐1‐en‐1‐yl)disulfane	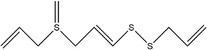	
29	Allyl(methyl)sulfane	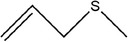	
30	1‐Allyl‐2‐methyldisulfane	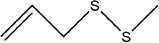	
31	(*E*)‐1‐allyl‐2‐(3‐(allylsulfinyl)prop‐1‐en‐1‐yl)disulfane	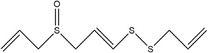	
32	(*Z*)‐1‐allyl‐2‐(3‐(allylsulfinyl)prop‐1‐en‐1‐yl)disulfane	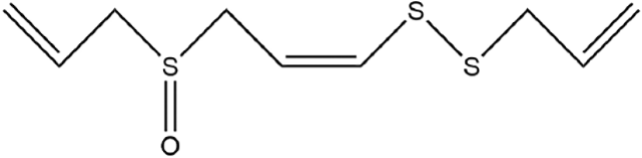	
33	3‐Vinyl‐3,4‐dihydro‐1,2‐dithiine	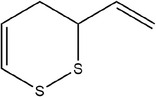	(Kim, Chun, et al., 2011; Kim, Park, et al., 2011)
34	1,3‐Diallyltrisulfane	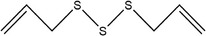	
35	1‐Allyl‐3‐methyltrisulfane	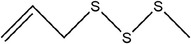	
36	1,4‐Diallyltetrasulfane	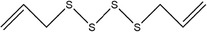	
37	1‐Allyl‐4‐methyltetrasulfane	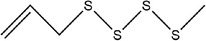	
38	2‐(Allylthio)‐2‐aminoacetic acid	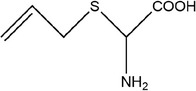	
39	(*E*)‐2‐allyl‐1‐methylene‐1‐(prop‐1‐en‐1‐yl)‐1λ^4^‐disulfane	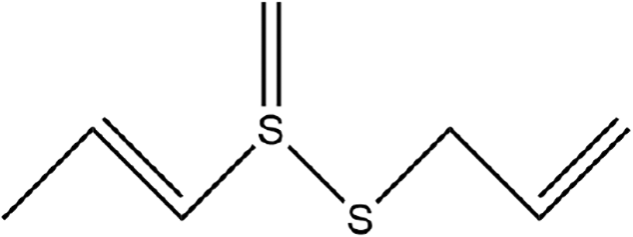	
40	(*E*)‐S‐(prop‐1‐en‐1‐yl) prop‐2‐ene‐1‐sulfinothioate	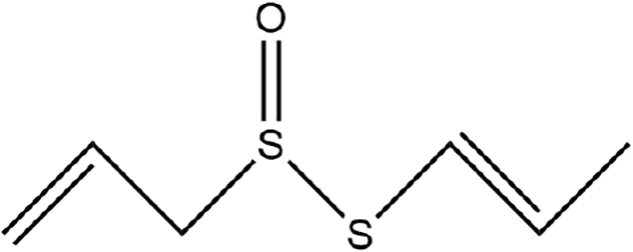	
41	*S*‐methyl prop‐2‐ene‐1‐sulfinothioate	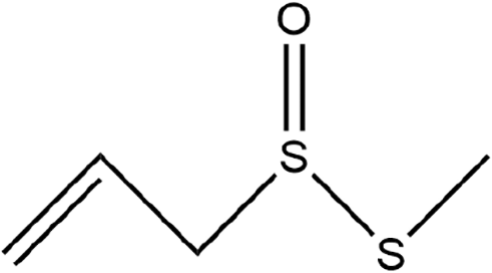	
42	*S*‐allyl methanesulfinothioate	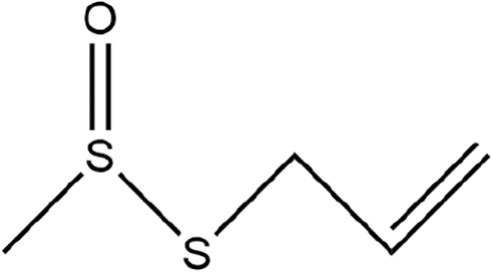	
43	*S*‐(allylthio)cysteine	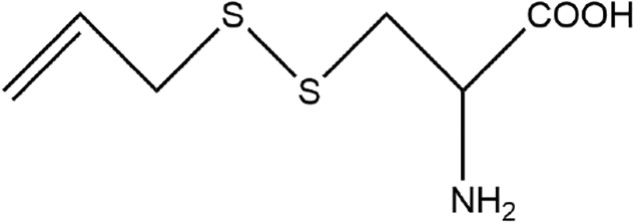	(Ichikawa et al., 2006; Miron et al., 2002)
44	(*E*)‐prop‐1‐ene‐1‐thiol	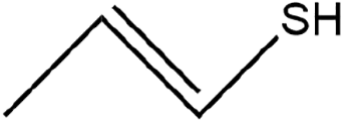	
45	2,2‐Dimethylthiirane	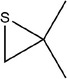	
46	Ethyl acetate	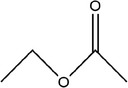	
47	Propylidene‐λ^4^‐sulfanone	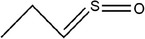	(Miron et al., 2002)
48	(*E*)‐1‐methyl‐2‐(prop‐1‐en‐1‐yl)disulfane	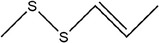	
49	1‐Methyl‐3‐propyltrisulfane	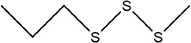	
50	Methyldimethylene‐λ^6^‐sulfanol	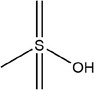	
51	Isopropyl (methyl) sulfane	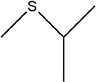	
52	Tetrahydrothiophene	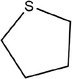	
53	1,2‐Dithiolane	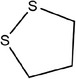	(Miron et al., 2002; Schulz et al., 1998)
54	3,4‐Dimethylthiophene	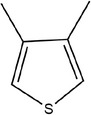	
55	*S*‐methyl methanesulfinothioate	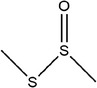	
56	*tert*‐butyl(methyl)sulfane	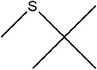	
57	Tetrahydro‐2*H*‐thiopyran‐3‐ol	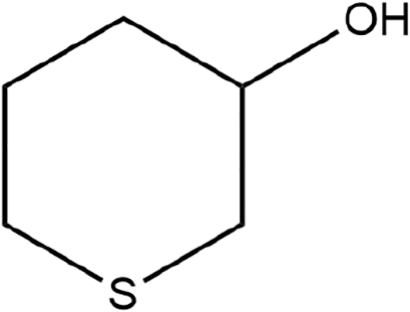	(Ichikawa et al., 2006; Schulz et al., 1998)
58	2‐Methyl‐1,3‐oxathiane	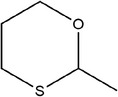	
59	3‐(Allylthio)propanoic acid	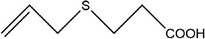	
60	3‐Methylene‐3,6‐dihydro‐1,2‐dithiine	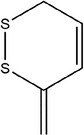	
61	3‐Methylene‐3,4‐dihydro‐1,2‐dithiine	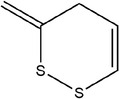	
62	(*Z*)‐1‐methyl‐3‐(prop‐1‐en‐1‐yl)trisulfane	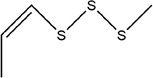	
63	Isobutyl(2‐methoxyallyl)sulfane	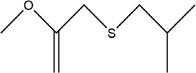	(Block et al., 1986; Schulz et al., 1998)
64	*S*‐methyl methanesulfonothioate	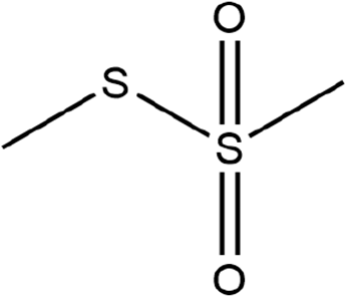	
65	Tetrahydro‐2*H*‐thiopyran‐4‐ol	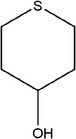	
66	Cyclopentyl(ethyl)sulfane	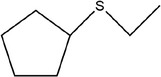	
67	Prop‐2‐ene‐1‐thiol	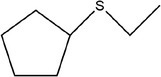	
68	Pyridine‐4‐thiol	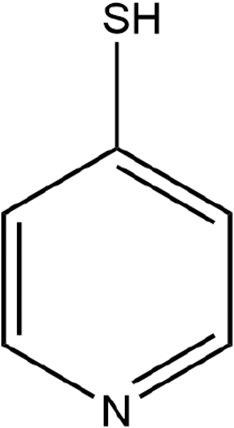	

Among the essential oils of garlic, thiosulfate is a more representative class of compounds. Thiosulfate exists in all *Allium* plants. These molecules are mainly converted from s‐alkyl‐l‐cysteine‐s‐oxide in the cytoplasm. The latter generates sulfite through a catalytic reaction of a C‐S lyase in the vesicles. These sulfites are usually highly reactive intermediates, which can immediately generate thiosulfite through condensation reaction in the cell (Alinezhad et al., 2013). Thiosulfates are very unstable compounds that undergo further rearrangements, resulting in a wide variety of derived sulfur compounds, which involve further transformations and remain biologically active. The most representative substances among sulfosuccinates are allyl compounds, which are also the most dominant in garlic essential oils.

## 3 BIOACTIVITIES OF GARLIC ESSENTIAL OIL

### 3.1 Antimicrobial activity

Since ancient times, people have been studying natural methods to prevent and treat diseases. Garlic has attracted extensive attention because of its beneficial properties and less side effects (Misharina et al., 2009).

**FIGURE 2:**
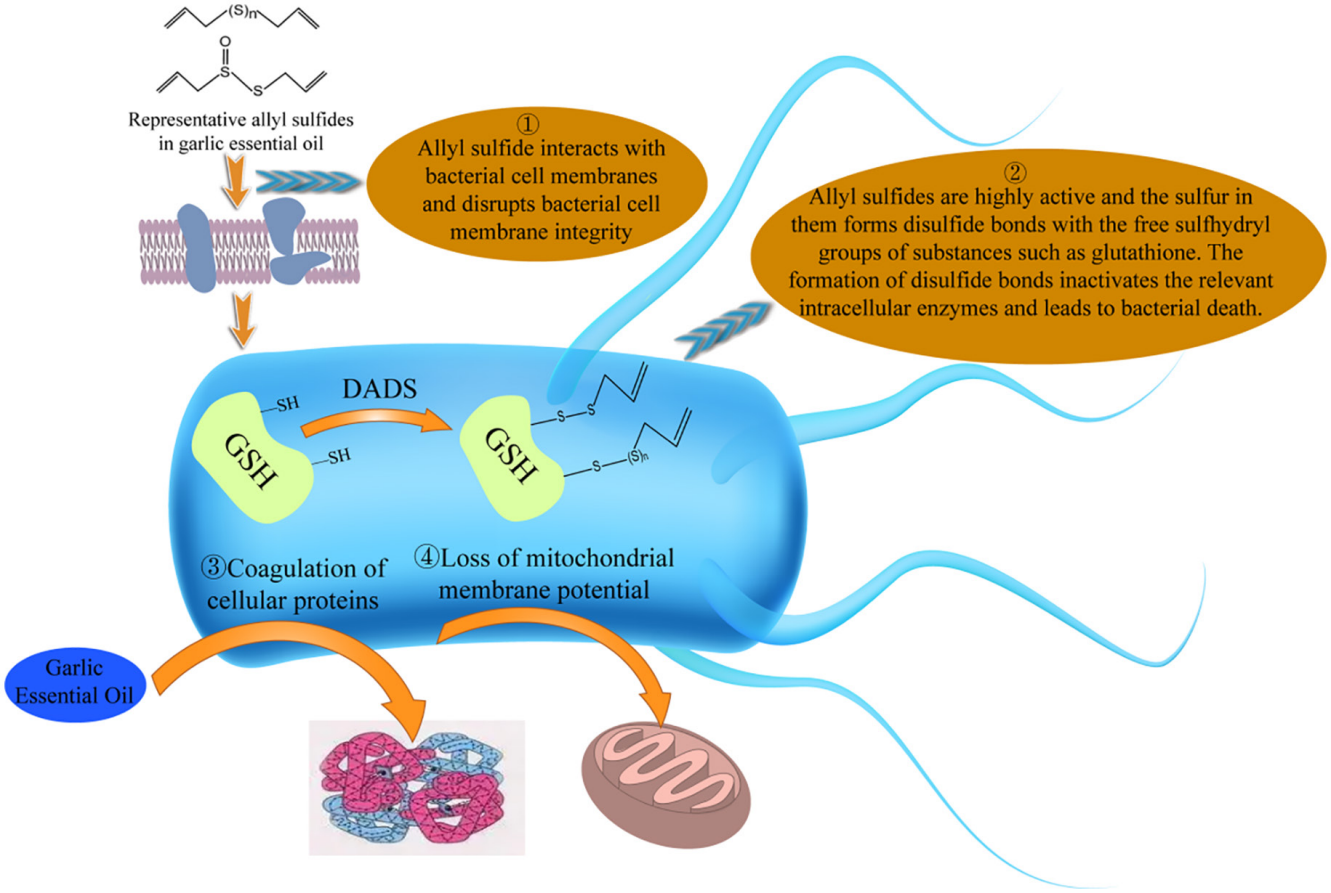
Mode of action of essential oils in bacteria.

Many plant essential oils have broad‐spectrum antibacterial, antifungal and antiviral activities. In addition, plant essential oil also showed strong growth inhibition for drug‐resistant strains that were difficult to be treated with traditional antibiotics. As for their mode of action, in fungal pathogens, plant essential oils destroy ATP assembly and cause cell wall damage by establishing membrane potential on the cell wall, resulting in fungal cell death. Plant essential oil can also play its antifungal role by destroying mitochondrial membranes and interfering with the electron transport system (de Turina et al., 2006). In bacterial pathogens, plant essential oil destroys the integrity of cell membrane, thus causing cell component leakage and ion loss, and affecting life activities such as energy production and membrane transport, resulting in the death of bacterial pathogen cells (Figure 2) (Burt, 2004).

Previous studies have shown that different solvent extracts of garlic essential oil (distilled water, methanol, and ethanol) can inhibit a variety of plant pathogens and fungi under different pH conditions (Chen et al., 2018). The water extract had the best antibacterial effect, and the antibacterial effect was the best under strong acid conditions (pH 3.0). Through scanning electron microscope observation, garlic essential oil extract may cause microbial cell death by destroying the structural integrity of the cell membrane, thus exerting its antibacterial effect. Studies have shown that the allyl sulfides in garlic essential oil exert their antibacterial effects by reacting with the sulfhydryl groups of many important metabolism‐related enzymes in bacteria (Kyung, 2012). The action of allicin is mostly non‐specific as it is found to inhibit urease, papain, amylase, and alcohol dehydrogenase. In the food industry, studies have found that chitosan films by incorporating garlic essential oil showed stronger growth inhibition than that of ordinary chitosan films against the food pathogens such as *Escherichia coli*, *Staphylococcus aureus*, *Salmonella typhimurium*, *Listeria monocytogenes*, and *Bacillus cereus* (Pranoto et al., 2015). Further studies revealed that the allyl sulfide in garlic essential oil could regulate the quorum sensing signal molecules and extracellular virulence factors of *Pseudomonas aeruginosa* in vivo and in vitro by suppressing the expression of *las*, *rhl*, and *pqs* (Cady et al., 2012; Persson et al., 2005).

The antibacterial activity of garlic essential oil is mainly derived from its abundant allyl sulfide and its derivatives, and the number of sulfur atoms determines the strength of its antibacterial ability (Casella et al., 2012; Tsao & Yin, 2001). In addition, it has been shown that allyl sulfide in garlic essential oil can exert its antibacterial activity by modulating genes related to bacterial metabolism, membrane transport system and secretion system. For example, in *Campylobacter jejuni*, allyl sulfide can downregulate 14 genes related to bacterial cellular homeostasis and oxidative stress transport proteins, thus reducing the ability of bacteria to adapt to adverse environments and the ability to adapt to adverse environments (Tang et al., 2021).

It has been found that DATS in garlic essential oil can regulate genes related to the membrane transport system and secretion system in bacterial pathogens. For example, in *C. jejuni* cells, DATS reduces the ability of bacteria to resist environmental stress by downregulating 14 genes related to environmental balance in bacterial cells. In addition, the amount of DATS in this study can also significantly affect the integrity of bacterial cell structure. Generally, treatment with high concentration of DATS can cause significant morphological changes such as cell deformation, dissolution, and cell membrane damage (Tang et al., 2021).

### 3.2 Antioxidant activity

It is well known that free radicals play an indispensable role in biological activities. Many endogenous and exogenous antioxidants have been successfully applied to treat cellular damage induced by NO. Although some synthetic antioxidants, such as butylhydroxyanisole and butylhydroxytoluene, are available, these synthetic antioxidants are accompanied by a range of side effects. Therefore, the recent trend in the control and treatment of diseases favors the use of natural compounds instead of synthetic ones to scavenge free radicals. The allyl sulfide that is rich in garlic essential oil has been shown to exert antioxidant effects through its ability to scavenge reactive oxygen species (ROS) (Borek, 2001). It can inhibit lipid peroxidation, reduce ischemic/reperfusion damage, and reduce oxidative stress, thereby protecting DNA from free radical–induced damage and mutations (Kim, Chun, et al., 2011; Kim, Park, et al., 2011; Shaarawy et al., 2009).

Garlic essential oil is usually obtained by distillation of chopped garlic. Compared with fresh garlic and other garlic derivatives, chopped garlic has higher antioxidant activity. This may be due to the subsequent extraction process enhancing stable and strongly bioactive water‐soluble organosulfur compounds, such as S‐allyl cysteine and S‐allyl mercapto cysteine, both of which possess strong antioxidant activity. Some of the allyl compounds in the essential oil of garlic are diallyl sulphide, DATS, DADS, and diallyl polysulfides, which exhibit antioxidant effects (Santhosha et al., 2013). It was found that some sulfur‐containing compounds in garlic essential oil could enhance the activities of superoxide dismutase (SOD), catalase (CAT), and glutathione peroxidase in cells and increase the level of glutathione in cells. Some of the important defense mechanisms in living cells promote the removal of ROS. Thus, they exert their antioxidant activity. It is worth mentioning that garlic polysaccharides could scavenge hydroxyl radicals and superoxide anions before and after phosphorylation (Chen et al., 2018).

It has been found that garlic essential oil can inhibit the lipid peroxidation induced by Fe NTA and reduce the level of glutathione and increase the production of H_2_O_2_. Garlic essential oil can also improve Fe‐NTA‐mediated renal injury by inhibiting the activities of antioxidant enzymes such as CAT, glutathione peroxidase, glutathione reductase, glucose‐6‐phosphate dehydrogenase, and glutathione S‐transferase. In addition, gallic essential oil can ameliorate diabetes‐induced oxidative stress in the rat liver model and normalize oxidative stress in alloxan‐induced diabetic rates (Iqbal & Athar, 1998; Saad Abdultawab & Ayuob, 2013).

The joint regulation of garlic essential oil and fish oil against oxidation and drug metabolism system also has been studied. Chen et al. found that with the increase of the dose of garlic essential oil, the activities of glutathione S‐transferase, glutathione reductase, SOD, and ethoxymethyl red *O*‐deethylase in the liver increased, while the activities of glutathione peroxidase and N‐nitrosodimethylamine demethylase decreased significantly (Chen et al., 2013). In addition, Mirunalini S et al. found that garlic essential oil plays a protective role in the peroxidation of liver and blood lipids during the carcinogenesis of hamster cheek pouch by regulating lipid peroxidation and enhancing the antioxidant status in liver and blood (Mirunalini et al., 2004).

Previous studies have shown that after allicin in garlic essential oil enters cells, it interacts with reducing glutathione to form S‐allyl mercaptoglutathione, which has a long‐term antioxidant effect (Horev‐Azaria et al., 2008; Rabinkov et al., 2000). Furthermore, allicin can also exert its antioxidant effect by positively regulating the KEAP1‐NRF2 regulatory pathway and reducing the transcription of cytoprotective genes (Hong et al., 2019; Li et al., 2012; Yang et al., 2020).

The antioxidant properties of garlic essential oil also have many applications in terms of drug side effects. For example, cyclophosphamide (CYP) is an alkylated anticancer drug with excellent effects. However, due to its off‐target multiple organ toxicity, its clinical use usually produces certain side effects, such as testicular injury (Mohammadi et al., 2013). Chima A. Ekeleme‐Egedigwe et al. investigated the effect of garlic essential oil on CYP‐induced oxidative stress and hormone deficiency in male rats, which tested by determining the total phenolic and total flavonoid content of garlic essential oil using DPPH and FRAP methods and measuring the antioxidant capacity of garlic oil (Ekeleme‐Egedigwe et al., 2020). The results showed that garlic essential oil significantly attenuated the reduction of testicular SOD, CAT, and glutathione peroxidase (GPx) activities induced by CYP. Furthermore, histopathology has confirmed that CYP can reduce the level of glutathione, significantly increase malondialdehyde (MDA), and significantly reduce the levels of serum testosterone, follicle‐stimulating hormone, and luteinizing hormone (Nafees et al., 2011). In contrast, garlic essential oil attenuates biochemical changes in the testes, increases hormone levels, reduces tissue damage, and protects the testes from CYP toxicity through its antioxidant properties. Zhang et al. did similar research. They found that garlic essential oil can inhibit N‐nitrosodiethylamine‐induced oxidative stress in rats by enhancing the levels of intracellular SOD, glutathione reductase, glutathione peroxidase, and glutathione‐S‐transferase, so as to achieve its antioxidant effect (Zhang, Zeng, et al., 2012).

The antioxidant properties of garlic essential oil also have many applications in water pollution treatment. For example, polluted water containing nanosilver is toxic to organisms because of toxic stress and tissue alterations. Khan et al. studied the toxicity of garlic essential oil on amine‐coated and spherical Ag‐NPs in freshwater rohu *Labeo rohita*. The results showed that garlic essential oil exerted its antioxidant activity by affecting the activities of SOD, CAT, and glutathione S‐transferase and reducing the content of lipid peroxidation and MDA (Khan et al., 2020).

### 3.3 Insecticidal activity

The growth of mosquito pests, structural pests, and economic pests in the world can seriously affect the growth of crops (Singh & Pandey, 2018). In addition, some parasites also pose a great threat to human health. Due to the shortcomings of the use of synthetic pesticides, the development of new biological pesticides has always been an important problem in the modern agricultural production system (Abdelgaleil et al., 2020). In recent years, some natural extracts have been found to have good insecticidal activity and can resist different pests and pathogens. For example, Zhang et al. used 97 essential oils such as garlic essential oil and black pepper oil to study their preventive and therapeutic effects on human Babesia disease caused by *Duncan Babesia* (Zhang, Bai, et al., 2020; Zhang, Liu, et al., 2020).

Sidiropoulou et al. found that garlic essential oil has a certain inhibitory effect on *Eimeria* in bovine kidney cells in vitro. At the same time, in vivo studies show that garlic essential oil can significantly reduce the number of intestinal bacteria and oocyst output. Therefore, it is concluded that garlic essential oil can be used as a significant inhibitor of *Eimeria* growth and reproduction (Sidiropoulou et al., 2020).

A similar study was carried out by Asghar et al. (2020). Their study evaluated the anti‐coccidial effects of garlic essential oil, ginger essential oil, and traditional anti‐coccidial drug Amprolium in vivo. The results showed that Amprolium and garlic essential oil had a good control effect on coccidiosis, but ginger essential oil had no clinical effect on quail coccidiosis (Sidiropoulou et al., 2020).

In addition, Kamel et al. studied the immunomodulatory effect of garlic essential oil on *Shistosomiasis mansoni* in mice at different developmental stages and its effect on enhancing the host immune system. The results showed that garlic essential oil could affect the larval and mature stages of the parasite, and enhanced the immune system of the host, so as to prevent and treat the diseases caused by schistosomiasis (Kamel & El‐Shinnawy, 2015).

In addition to its inhibitory effect on pests, studies have shown that allyl disulfide in garlic essential oil can also prevent pests by inhibiting the hatching of insect eggs. Muturi et al. further studied the toxicity of Garlic essential oil and asafoetida essential oil to *Culex pipiens Linnaeus* and its larvae when used alone or in combination. The results showed that allyl disulfide in the two essential oils showed strong egg killing and larvicidal activities, indicating that it played an important role in the overall toxicity of the two essential oils (Muturi et al., 2018).

### 3.4 Medical relevance

The health benefits of garlic essential oil in the treatment of various diseases have been widely studied in animals and humans, such as anti‐cancer, anti‐inflammatory, anti‐diabetes, anti‐hyperlipidemia, liver protection, anti‐fibrinolysis, and anti‐platelet aggregation activities, as well as its potential role in the prevention of cardiovascular diseases. The effects of some allyl sulfides in garlic essential oil on cells are listed in Table 2.

**TABLE 2:** Effects of some allyl sulfides in garlic essential oil on cells.

Object of action	Objectives	Primary outcome	References
Madin‐Darby canine kidney cells	The early signaling effects of diallyl sulfide on renal cells loaded with Ca^2+^‐sensitive dye fura‐2	Diallyl sulfide induced a significant rise in [Ca^2+^]_i_ in Madin‐Darby canine kidney renal tubular cells by stimulating both extracellular Ca^2+^ influx and thapsigargin‐sensitive intracellular Ca^2+^ release	(Chen et al., 2009)
Kupffer cell in mice	Explore whether the protective effect of diallyl sulfide on lipopolysaccharide /D‐galactosamine‐induced acute liver injury was associated with the regulation of Kupffer cell activation	The protective effects of diallyl sulfide on lipopolysaccharide/D‐galactosamine induced acute liver injury may be achieved by inhibiting the activation of the Kupffer cell, thereby suppressing the secretion of various proinflammatory mediators	(Li et al., 2019)
Rat colonocytes	The effects of DADS on histone H4 and H3 acetylation levels in vivo in colonocytes isolated from non‐tumoral rat	The involvement of histone acetylation in modulation of gene expression by DADS in normal rat colonocytes, which might play a role in its biological effects as well as in its anticarcinogenic properties in vivo	(Druesne‐Pecollo et al., 2007)
Neuroblastoma cells	Modulation of peroxisomes in Neuroblastoma cells by DADS	Garlic‐derived DADS is able to increase PGC1a expression in a ROS‐dependent manner and to induce mitochondrial biogenesis at early stage of treatment	(Pagliei et al., 2013)
Human colon tumor cells	Effects of DADS on arylamine N‐Acetyltransferase activity in human colon tumor cells	DADS has an inhibitory effect on arylamine N‐acetyltransferase activity in a human colon tumor cell line	(Chen et al., 1998)
COLO 205 human colon cancer cells	Effects of diallyl polysulfides(diallyl sulfide, DADS and DATS) on the gene expression of the multidrug resistance in colo 205 human colon cancer cells in vitro and in vivo	Diallyl sulfide, DADS and DATS presented different effects on drug‐resistant gene expression levels in colo 205 cells in vitro and in vivo. DATS has greater stimulatory effects on drug resistance gene expression levels but cytotoxicity in vitro and in vivo is higher for DATS than that of diallyl sulfide and DADS	(Lai et al., 2012)
Human A549 lung tumor cells	The antiproliferative effects of DATS and DADS on cultured human neoplastic (A549) and nonneoplastic (MRC‐5) lung cells	DATS is extremely effective in retarding the proliferation of cultured human lung tumor cells. Neoplastic cells (A549) were considerably more sensitive than nonneoplastic (MRC5) human lung cells to DATS, the response is obviously dose dependent	(Sakamoto et al., 1997)
Human melanoma A375 cells and basal cell carcinoma cells	Mechanism of action of diallyl sulfide, DADS and DATS on skin cancer cells	DATS increased intracellular ROS generation, induced cytosolic Ca2þ mobilization, and decreased mitochondrial membrane potential	(Wang, Qin, et al., 2010; Wang, Yang, et al., 2010)
Human hepatoma (HepG2) cells	Determine whether the mechanism of biological action of garlic‐derived sulfur compounds in human hepatoma (HepG2) cells can be dependent on the presence of labile sulfane sulfur in their molecules	DATS showed the highest biological activity in HepG2 cells. DATS did not affect the activity of sulfurtransferases and lowered sulfane sulfur level in HepG2 cells	(Iciek et al., 2011)

#### 3.4.1 Antitumor activity

Cancer, also known as a malignant tumor, kills up to 7 million people worldwide every year. At present, the methods to treat cancer include surgery, radiotherapy, and chemotherapy, but most of them will lead to many side effects after treatment. Therefore, the search for new natural alternative therapies has become an important direction in the prevention and treatment of cancer. Natural products from plants have attracted much attention in recent years because of their excellent antitumor activity and less side effects (De Greef et al., 2021).

Lan et al. detected the cell cycle and apoptosis of pancreatic cancer cells after the action of garlic essential oil by flow cytometry, staining with propidium iodide and annexin V‐fluorescein isothiocyanate. The morphological changes of pancreatic cancer cells were observed by transmission electron microscope. The results showed that garlic essential oil significantly inhibited the proliferation of pancreatic cancer cells. In addition, garlic essential oil induced apoptosis of pancreatic cancer cells through programmed cell death or cell cycle arrest in vitro, thus exerting its antitumor activity. With the increase of garlic essential oil dosage and the prolongation of action time, the effect of inducing apoptosis of pancreatic cancer cells was more obvious (Lan et al., 2013). Similarly, previous studies have confirmed the role of oxidative stress and its association with tumor promotion. Researchers believe that garlic essential oil can be used as an effective chemopreventive agent to inhibit tissue damage and carcinogenesis induced by oxidants and protect liver toxicity induced by Fe‐NTA (Agarwal et al., 2007).

In addition to its strong anticancer activity, garlic essential oil can also be used to inhibit various sequelae after cancer treatment. Zeng et al. established a tumor xenograft mice model by subcutaneous injection of H22 tumor cells and investigated the effects of garlic essential oil on chemo/radiotherapy. They found that garlic essential oil could not enhance the therapeutic effect of radiotherapy and chemotherapy on tumors. But the reduction of the endogenous spleen colonies induced by chemo/radiotherapy was significantly suppressed by garlic essential oil cotreatment (Zeng et al., 2013).

At present, the anticancer mechanism of DATS in garlic essential oil is not completely clear, but its pharmacological reactions are known to include the change of carcinogenic metabolic enzymes, cell cycle arrest, induction of apoptotic cell death, inhibition of carcinogenic signal transduction pathway, and inhibition of neoangiogenesis (Antony & Singh, 2011). The suppression of cytochrome P450‐dependent monooxygenases is considered to be the main mechanism of cancer chemoprevention through naturally occurring dietary bioactive compounds. Sakamoto et al. first proved the anti‐proliferation effect of DATS in garlic essential oil on cancer cells (Sakamoto et al., 1997). DATS‐mediated cancer cell proliferation inhibition is related to cell cycle arrest, including human hepatoma cells (Wu et al., 2004), gastric cancer cells (Ha et al., 2005), colon cancer cells (Hosono et al., 2005), prostate cancer cells (Herman‐Antosiewicz & Singh, 2005; Xiao et al., 2005; Xiao, Zeng, Hahm, et al., 2009; Xiao, Zeng, & Singh, 2009), lung cancer cells (Wu et al., 2009; Xiao, Zeng, Hahm, et al., 2009; Xiao, Zeng, & Singh, 2009), bladder cancer cells, and skin cancer cells (Wang, Qin, et al., 2010; Wang, Yang, et al., 2010). DATS treatment leads to the degradation of ferritin and the increase of labile iron levels, resulting in the production of ROS. Cdc25C was inhibited by N‐acetylcysteine and other antioxidants, and DATS‐induced ROS production in cells, which further led to the downregulation of Cdc25C. At present, it has been found that DATS can achieve the therapeutic purpose by downregulating the expression of Cdk1 in prostate cancer cells, but the specific mechanism is not clear (Wang, Qin, et al., 2010; Wang, Yang, et al., 2010; Xiao, Zeng, Hahm, et al., 2009; Xiao, Zeng, & Singh, 2009).

**FIGURE 3:**
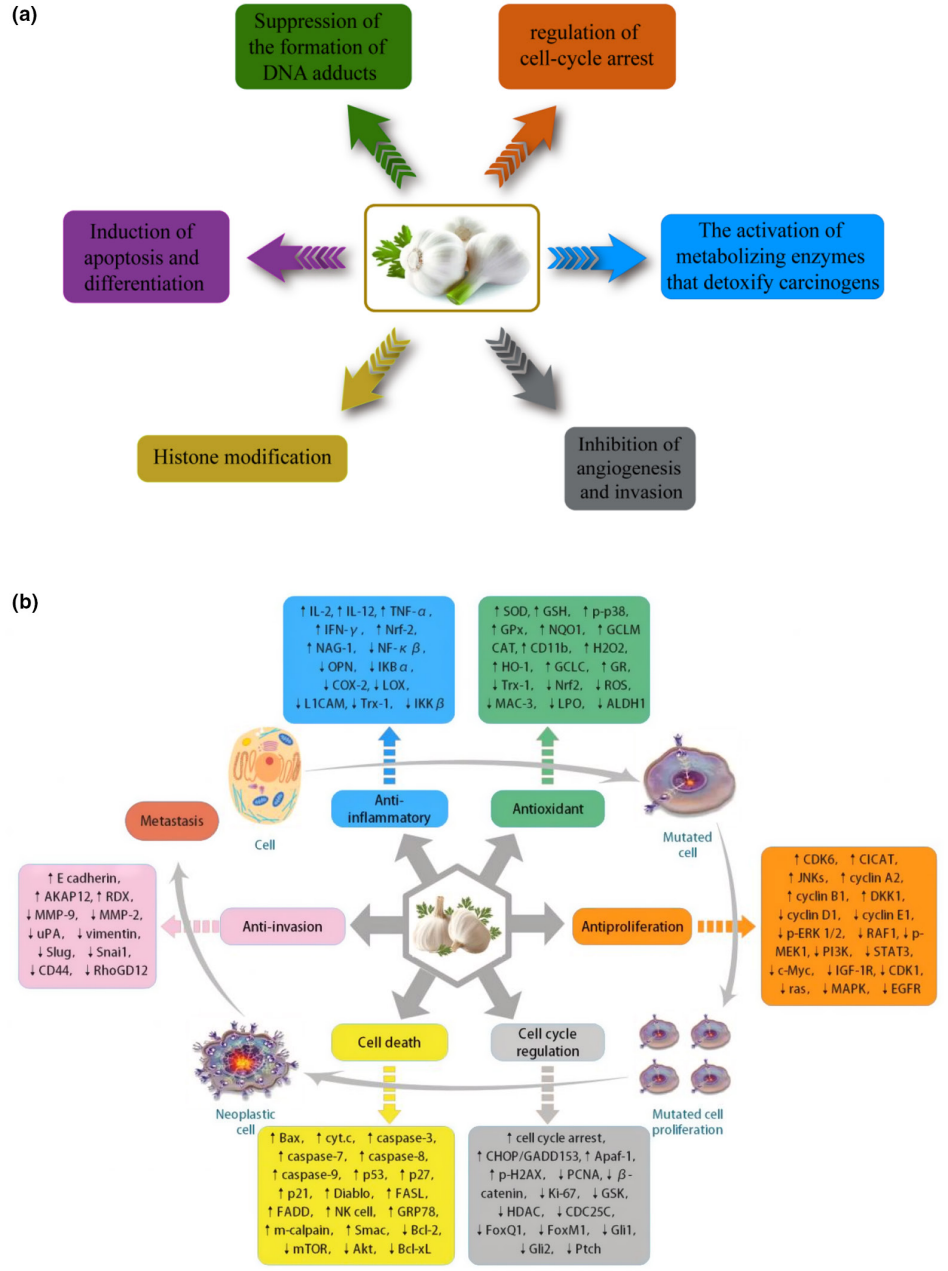
(a) Major antineoplastic mechanisms of active ingredients of garlic essential oil. (b) Major molecular targets, and signaling pathways of active ingredients of garlic essential oil.

The active components in garlic essential oil usually affect the life activities of tumor cells by regulating various signal pathways and genes involved in tumor genesis, invasion, and metastasis. In different stages of cell carcinogenesis, the active components of garlic essential oil usually exert their anticancer effects through different active effects. These include anti‐oxidation and anti‐inflammatory effects on primary cancer cells, anti‐proliferation and cell cycle regulation on mutant cells, induction of apoptosis on tumor cells and anti‐invasion during tumor cell proliferation and metastasis. Figure 3 shows the main anti‐tumor mechanisms, molecular targets, and signal pathways of the active components of garlic essential oil.

#### 3.4.2 Anti‐inflammatory activity

Inflammation is a kind of defense response of the body to stimulation, which is characterized by redness, swelling, heat, pain and dysfunction. Usually, inflammation is beneficial and an automatic defense response of the human body, but sometimes, inflammation is also harmful, such as attacks on human tissues and inflammation in transparent tissues. Similarly, excessive or persistent inflammation can lead to asthma, conjunctivitis, arthritis, and other diseases. At present, natural products have attracted researchers' interest because of their excellent anti‐inflammatory activity and almost no side effects.

Many previous studies have proved that the good anti‐inflammatory activity of garlic essential oil is also achieved through its antioxidant effect. Its main mechanism is to inhibit the enzymes involved in the production of inflammatory prostaglandins and thromboxanes, inhibit the activation of NFkB and the expression of pro‐inflammatory cytokines and inducible NO synthase (Sener et al., 2007). In inflammatory bowel diseases, garlic essential oil exerts anti‐inflammatory effects through dysregulation of IL‐10 and reduction of IL‐12, preventing IL‐12 from binding to its receptors on T and NK cells and, thus, inhibiting IFN‐γ production (Hodge et al., 2002). In addition, some sulfides in garlic essential oil exerted their anti‐inflammatory effects by inhibiting the production of NO, prostaglandin E2, and the expression of the pro‐inflammatory cytokines tumor necrosis factor, interleukin‐1b, and interleukin‐6 in lipopolysaccharide‐activated macrophages (Lee et al., 2012).

The anti‐inflammatory effect and potential molecular mechanism of DADS in garlic essential oil on acute pancreatitis and related lung injury in mice have also been studied (Mathan Kumar & Tamizhselvi, 2020). The researchers found that DADS can significantly reduce the level of related enzymes in mouse pancreatic cells, reduce the production of H_2_S, affect tumor necrosis factor TNF‐ α, and inhibit the infiltration of macrophages and neutrophils. In addition, DADS reduced caerulein‐induced I‐κB degradation and subsequent translocation of NF‐κB in the pancreas and lung.

Similarly, garlic essential oil is also used to treat peptic ulcers due to its antioxidant properties. Previous studies have found that intracellular glutathione can protect gastric mucosal cells from ethanol‐induced damage in vivo and in vitro (Mutoh et al., 1990). In a study by Rodrigues et al., terpenes and their derivatives have been shown to have gastric protective activity and significantly increase the concentration of glutathione in adenocarcinoma gastric cells (Rodrigues & Percival, 2019). The same results were confirmed by the study of Kuna et al. (2022). Moreover, Stagos et al. found that quercetin, a plant polyphenol in garlic essential oil, significantly increased the level of glutathione in vivo and in vitro and inhibited the decrease of glutathione (Stagos et al., 2012). El‐Ashmawy et al. found that garlic essential oil can reduce oxidative stress caused by gastric ulcer anti‐inflammatory drugs and reduce glutathione consumption (El‐Ashmawy et al., 2016).

#### 3.4.3 Anti‐diabetic and anti‐hyperlipidemic activity

Diabetes mellitus is a metabolic disorder caused by insufficient insulin secretion and insulin antagonism, characterized by persistent hyperglycemia, which eventually leads to specific complications. According to Ryan et al., about one‐third of people with diabetes choose to use alternative medications, which they believe are effective, and garlic is the most commonly used of these alternatives (Ryan et al., 2001). However, there are few studies related to the hypoglycemic effect of the active ingredients in garlic essential oil and some researches are inconsistent. In the 1970s, Jain et al. studied the hypoglycemic effect of aqueous extracts of garlic as well as several different organic solvent extracts on normal and alloxan‐induced diabetic rabbits (Jain et al., 1973). They found all of the garlic preparations had acute hypoglycemic effects, with the action of the ether extract being competitive with that of tolbutamide, a drug used to treat diabetes. Meanwhile, Duncan et al. found in diabetic patients, garlic essential oil can improve the symptoms of hyperglycemia (Duncan, 1999). In addition, the study found that S‐allylcysteine sulfoxide, the precursor of DADS in garlic essential oil, also has hypoglycemic effect, and the mode of action is similar to that of glibenclamide, a traditional hypoglycemic drug (Sheela & Augusti, 2002).

In the skeletal muscle cells of streptozotocin‐induced diabetes rats, the content of glucose transporter‐4 and glucose transport rate were lower, and the activity of glycogen synthase was lower, which further reduced the glucose utilization stimulated by insulin and lactate production in the cells (Muñoz et al., 1996; Oku et al., 2000). The DATS in garlic essential oil improves glycemic control in diabetic rats through increased insulin secretion and increased insulin sensitivity (Liu et al., 2005).

In addition, it has been shown that the DADS in garlic essential oil can improve the level of phosphodiesterase 5, a negative regulator of cyclic guanosine monophosphate(cGMP), which was upregulated in adipose tissues of high‐fat/high‐sucrose (HF/HS) diet‐fed mice (Liu et al., 2006). Also, DADS is thought to suppress the HF/HS diet‐induced upregulation of fatty acid synthesis‐related enzymes including sterol regulatory element‐binding protein‐1, fatty acid synthase, and stearoyl‐CoA desaturase‐1 (Bae et al., 2018).

Previous research found that garlic essential oil significantly enhanced SOD activity in liver and kidney tissue homogenates of streptozotocin‐induced diabetic rats, and garlic essential oil may effectively normalize the impaired antioxidants status in streptozotocin‐induced diabetes (Anwar & Meki, 2003). Garlic essential oil may be used in delaying the complicated effects of diabetes like retinopathy, nephropathy, and neuropathy due to an imbalance between free radicals and antioxidant systems. In similar studies, Liu et al. found that garlic essential oil can improve the glucose utilization stimulated by insulin in vivo or in vitro, so as to synthesize glycogen in skeletal muscle, and then play its role in the treatment of streptozotocin‐induced diabetes (Liu et al., 2012). The possible reason is that garlic essential oil increased glutathione peroxidase activity and decreased nitrate level in skeletal muscle cells of diabetes rats.

Bae J et al. have shown that cyclic guanosine monophosphate is an important mediator of EGCG ((−)epigallocatechin‐3‐O‐gallate), and garlic essential oil has a strong inhibitory effect on the negative regulator of cyclic guanosine monophosphate (phosphodiesterase) (Bae et al., 2017). In a follow‐up experiment, they found an increase in the content of cyclic guanosine acid negative regulator phosphodiesterase in the adipose tissue of mice fed an HF/HS diet, which in turn was ameliorated by DADS, which is the major organosulfur compound in garlic essential oil. They also found that the combination of green tea extract and DADS attenuated HF/HS diet‐induced increase in hepatic fat and triglyceride accumulation. Finally, they concluded DADS in garlic volatile oil enhanced the anti‐obesity effect of green tea extract while inhibiting sterol regulatory element‐binding protein‐1 and activating peroxisome proliferator‐activated receptors (Bae et al., 2018).

In addition to DADS, DATS is another major component of garlic essential oil. Diabetic rats were fed with garlic essential oil (100 mg/kg body weight), diallyl three thioethers (40 mg/kg body weight), and corn oil every other day for three consecutive days. The effects of garlic essential oil and diallyl three thioether on blood glucose control in streptozotocin‐induced diabetic rats were studied (Liu et al., 2005). The results showed that garlic essential oil and DATS improved glycemic control in diabetic rats by increasing insulin secretion and insulin sensitivity.

#### 3.4.4 The hepatoprotective effects

At present, the research on the liver protection of garlic essential oil mainly focuses on allyl sulfide. DADS and DATS are the main active components in garlic essential oil. It is reported that both of them have certain liver and kidney protective activities (Avato et al., 2000).

The molecular mechanisms of DADS and diallyl sulfide that induced hepatocyte toxicity was investigated by Truong et al. They found that compared with allyl sulfide, NaHS had the greatest cytotoxicity on hepatocytes, followed by DADS, and DATS had the least effect. All three compounds can reduce the mitochondrial membrane potential of hepatocytes and increase the formation of ROS and thiobarbituric acid reactive species (TBARS) (Truong et al., 2009). Eventually, it was concluded that, on the one hand, DADS‐induced cytotoxicity was prevented by the H_2_S scavenger hydroxocobalamin, which also prevented cytochrome oxidase‐dependent mitochondrial respiration suggesting that H_2_S inhibition of cytochrome oxidase contributed to DADS hepatocyte cytotoxicity. In addition, studies have shown that DADS can accelerate the consumption of GSH, reduce mitochondrial membrane potential, and increase the formation of ROS and TBARS in liver and kidney cells, while hydralazine can inhibit this process.

In terms of hepatocyte toxicity, traditional therapeutic drugs acetaminophen (AAP) have certain side effects (Latchoumycandane et al., 2007). Excess AAP leads to excessive production of NAPQI, which in turn depletes GSH levels in hepatocytes, leading to subsequent hepatocyte death (Zanger & Schwab, 2013). Recent studies have demonstrated that the production of ROS induced by AAP causes mitochondrial damage and early activation of mitogen‐activated protein kinases, especially c‐Jun‐N‐terminal protein kinase (JNK). Ko et al. found through experimental findings that pretreatment with DADS in garlic essential oil effectively attenuated acute liver injury and oxidative stress caused by AAP. At the same time, DADS pretreatment also inhibited cytochrome P450 2E1 (CYP2E1) levels and suppressed the increased activity of CYP2E1 which was induced by AAP. It was finally concluded that DADS had a protective effect against AAP‐induced acute hepatotoxicity (Ko, Park, et al., 2017; Ko, Shin, et al., 2017). The study of El‐Khayat et al. also proved the protective effect of garlic essential oil on experimental animal liver injury (Zakaria et al., 2010). Similar to hepatocytes, in another experimental study, Ko et al. found DADS had a similar effect in mouse kidney cells (Ko, Park, et al., 2017; Ko, Shin, et al., 2017). Furthermore, Somade OT et al. found that the protective effect of DADS in garlic essential oil against trichloromethane (CHCl_3_)‐induced nephrotoxicity (Somade et al., 2018).

## 4 TOXICITY STUDIES ON GARLIC ESSENTIAL OIL

Garlic has been widely used in food and medicine since ancient times. Therefore, it is generally recognized that garlic, as a common food, is harmless to the human body (Brodribb, 2018). At present, there are not many in‐depth studies on the toxicity of garlic essential oil. Previous studies have found that the use of excessive garlic essential oil will stimulate the eye mucosa, and then cause certain damage to the normal function of the eyes. In serious cases, it may cause adverse consequences such as blurred vision and dry eyes. In addition, excessive consumption of garlic essential oil is likely to damage the gastrointestinal function of the human body. This is because excessive consumption of garlic essential oil will damage the red blood cells in the human body and cause strong stimulation to human gastric mucosa. If the consumption is not controlled in time, anemia or various gastrointestinal diseases will be caused.

It is found that the LD_50_ of DADS in female and male mice is 130 mg/kg and 140 mg/kg, respectively (Amagase, 2006). DADS in garlic essential oil can adversely affect the signal pathways related to the growth and proliferation of nerve cells in the hippocampus. DADS does not affect the life activities of mature nerve cells but can affect neural progenitor cells (Ji et al., 2013).

In addition, it has been shown that some of the volatile components of garlic essential oil may cause some acute toxicity, such as stomach ulcers, nausea, and vomiting (de Muniz et al., 2021). Excessive intake of garlic oil may also cause a number of chronic diseases, such as renal hematoma. Excessive intake of garlic oil can also increase the risk of miscarriage in pregnant women. There are limited studies on the toxicity of garlic essential oil. Previous studies have speculated that the fat‐soluble sulfur‐containing compounds in garlic essential oil are more toxic than the water‐soluble fraction, but specific evidence from experiments is still needed to verify this conclusion (Rahman, 2007).

## 5 CONCLUSIONS

Garlic essential oil has been reported as a frequently used natural product and has several potential health benefits. In this review, composition, biological activity, effect on human health, and application in the medical field of garlic essential oil are comprehensively summarized. Pharmacological findings give some help to the industry of functional food ingredients and formulation of new drugs. In animal experiments and clinical trials, it has been proved that garlic essential oil may be considered as a new drug to prevent cancer, oxidative damage, and inflammation, protect the liver, and reduce blood sugar. Meanwhile, garlic essential oil can inhibit or alleviate the side effects induced by some existing drug treatments. Furthermore, garlic essential oil can be used to overcome environmental pollution caused by microorganisms, control agricultural pests, and produce bioactive natural products.

Although great progress has been made in the research of garlic essential oil, there are still some problems to be studied and solved in the molecular mechanism of biological activity. For example, there are few studies on anti‐neuroinflammation and the mechanism of garlic essential oil. According to previous reports, garlic essential oil has shown effective therapeutic options against several diseases like cancer, diabetes, inflammation, and hepatoprotective activity. To develop ideal therapeutic drugs, it is necessary to study the pharmacology of garlic essential oil in vivo and in vitro. Future research should explore the toxicity, clinical therapeutic effect, and clinical side effects of garlic essential oil and clarify its active mechanism at the molecular, cellular, and gene levels.

## FUNDING INFORMATION

The authors did not receive support from any organization for the submitted work.

## CONFLICT OF INTEREST STATEMENT

None declared.

## ETHICS STATEMENT

Not applicable.

## Data Availability

Data sharing is not applicable to this article as no data sets were generated or analyzed during the current study.
